# Apoplexy in sporadic pituitary adenomas: a single referral center experience and AIP mutation analysis

**DOI:** 10.20945/2359-3997000000358

**Published:** 2021-04-27

**Authors:** Christhiane Fialho, Monique Álvares Barbosa, Carlos Henrique Azeredo Lima, Luiz Eduardo Armondi Wildemberg, Mônica R. Gadelha, Leandro Kasuki

**Affiliations:** 1 Universidade Federal do Rio de Janeiro Hospital Universitário Clementino Fraga Filho Faculdade de Medicina Rio de Janeiro RJ Brasil Centro de Pesquisas em Neuroendocrinologia/Seção de Endocrinologia, Faculdade de Medicina e Hospital Universitário Clementino Fraga Filho, Universidade Federal do Rio de Janeiro, Rio de Janeiro, RJ, Brasil.; 2 Instituto Estadual do Cérebro Paulo Niemeyer Unidade de Radiologia Rio de Janeiro RJ Brasil Unidade de Radiologia, Instituto Estadual do Cérebro Paulo Niemeyer, Secretaria Estadual de Saúde, Rio de Janeiro, RJ, Brasil.; 3 Instituto Estadual do Cérebro Paulo Niemeyer Laboratório de Neuropatologia e Genética Molecular Rio de Janeiro RJ Brasil Laboratório de Neuropatologia e Genética Molecular, Instituto Estadual do Cérebro Paulo Niemeyer, Secretaria Estadual de Saúde, Rio de Janeiro, RJ, Brasil.; 4 Instituto Estadual do Cérebro Paulo Niemeyer Unidade de Neuroendocrinologia Rio de Janeiro RJ Brasil Unidade de Neuroendocrinologia, Instituto Estadual do Cérebro Paulo Niemeyer, Secretaria Estadual de Saúde, Rio de Janeiro, RJ, Brasil.; 5 Hospital Federal de Bonsucesso Seção de Endocrinologia Rio de Janeiro RJ Brasil Seção de Endocrinologia, Hospital Federal de Bonsucesso, Rio de Janeiro, RJ, Brasil.

**Keywords:** Apoplexy, pituitary adenomas, *AIP*, familial isolated pituitary adenomas

## Abstract

**Objective:**

To analyze the clinical, laboratory, and radiological findings and management of patients with clinical pituitary apoplexy and to screen for aryl hydrocarbon receptor-interacting protein (AIP) mutations.

**Subjects and methods:**

The clinical findings were collected from the medical records of consecutive sporadic pituitary adenoma patients with clinical apoplexy. Possible precipitating factors, laboratory data, magnetic resonance imaging (MRI) findings and treatment were also analyzed. Peripheral blood samples were obtained for DNA extraction from leukocytes, and the entire AIP coding region was sequenced.

**Results:**

Thirty-five patients with pituitary adenoma were included, and 23 (67%) had non-functioning pituitary adenomas. Headache was observed in 31 (89%) patients. No clear precipitating factor was identified. Hypopituitarism was observed in 14 (40%) patients. MRI from 20 patients was analyzed, and 10 (50%) maintained a hyperintense signal in MRI performed more than three weeks after pituitary apoplexy (PA). Surgery was performed in ten (28%) patients, and 25 (72%) were treated conservatively with good outcomes. No AIP mutation was found in this cohort.

**Conclusion:**

Patients with stable neuroophthalmological impairments can be treated conservatively if no significant visual loss is present. Our radiological findings suggest that hematoma absorption lasts more than that observed in other parts of the brain. Additionally, our study suggests no benefits of AIP mutation screening in sporadic patients with apoplexy.

## INTRODUCTION

Pituitary apoplexy (PA) is an acute event associated with hemorrhage or infarction and occurs in approximately 2% to 12% of those with a preexisting pituitary adenoma (1–4). Sudden, abrupt and intense headache, visual impairment, an altered level of consciousness and clinical manifestations of hypopituitarism are described in the acute onset of PA (2–6).

The pathophysiology of apoplexy involves changes in the pituitary blood supply and can be related to rapid tumor enlargement that increases metabolic demand and intrasellar pressure, leading to the compression of adjacent structures (7). Therefore, PA occurs mostly in macroadenomas (8–10). Similarly, nonfunctioning pituitary adenomas (NFPAs) are the most prevalent in a series of apoplexy, likely related to silent growth, identified only after the development of a mass effect (3,9,11).

Risk factors for PA have not been completely elucidated, and potential precipitating factors were identified in 10% to 40% of cases, including angiography procedures, cardiac and other major surgeries, dynamic pituitary function tests, arterial hypertension, radiation therapy, head trauma, anticoagulation and antiplatelet therapy (2,9,12–14). Estrogen therapy, coagulopathies and intense exercise were also reported. Some case reports described PA during the treatment of acromegaly with octreotide (15).

Computed tomography (CT) is generally the first imaging tool available in the emergency room and can detect pituitary expansive masses in up to 80%-94% of cases, but a PA diagnosis is made in only 21% to 28% of cases (6,15,16). Magnetic resonance imaging (MRI) is the better choice for image analysis, confirming the diagnosis of PA in approximately 90% of patients (3,6,8,16).

No consensus exists concerning the best PA approach, and treatment can be conservative or surgical according to each case and condition (16,17). Some studies have demonstrated a good response in visual recovery with conservative management, considering selected patients with non-progressive neuroophthalmological deficits (6,18,19). During the acute phase, all the patients with clinical findings of suspected apoplexy must be tested for hypopituitarism, mainly because of the risk of severe hypocortisolism (2).

Interestingly, some series described PA as a feature of patients harboring germline mutations in the *aryl hydrocarbon receptor-interacting protein* (*AIP*) gene (20–22). Typically, patients with *AIP* mutations (*AIP*muts) have macroadenomas of young onset and aggressive behavior (20,21,23–25). *AIP*muts are present in approximately 20% of familial isolated pituitary adenoma (FIPA) kindreds and in 3.6% to 20% of apparently sporadic adenomas varying according to the age of the group analyzed, but mutations have low penetrance in both groups (21,22,26–28). To date, the frequency of *AIP*muts in patients with apparently sporadic pituitary adenomas presenting with PA is unknown.

Our study analyzed the clinical, radiological and therapeutic characteristics of patients who presented with clinical PA and were referred to a specialized neurosurgery center. We also evaluated the frequency of *AIP*muts in these apparently sporadic pituitary adenoma patients.

## SUBJECTS AND METHODS

### Patients

We retrospectively analyzed the files in our database from consecutive patients referred to a neurosurgery center [*Instituto Estadual do Cérebro Paulo Niemeyer* (IECPN)] with a clinical history and MRI report of PA from August 2013 to September 2017. We included patients who presented with sudden onset of severe headache and/or other neuroophthalmological symptoms (visual disturbance and/or ophthalmoplegia and/or altered consciousness) diagnosed with PA according to the UK Guidelines for the Management of Pituitary Apoplexy and who had undergone MRI to confirm PA (2). Headache was classified as thunderclap headache when it was described as bilateral and retroocular, with abrupt onset associated with progression to maximum intensity within a few minutes (29).

Blood samples were collected from the patients for genetic analysis (performed in our laboratory). At that time, they were interviewed to clarify all the symptoms presented at the acute phase. On admission, visual fields were assessed by confrontation tests and Goldmann manual campimetry.

Patients with clinical features and/or a history of X-linked acrogigantism (XLAG), multiple endocrine neoplasia type 1 (MEN-1) and 4 (MEN-4), Carney complex (CNC), association of pheochromocytoma/paraganglioma and pituitary adenoma syndrome (3PAs) and FIPA were excluded. All the subjects signed written informed consent, and the Ethics Committee of Medical School and *Hospital Universitário Clementino Fraga Filho* (HUCFF) of *Universidade Federal do Rio de Janeiro* (UFRJ) approved the study.

## METHODS

### Laboratory analyses

We collected the serum basal levels of growth hormone (GH), insulin-like growth factor type I (IGF-I), prolactin (PRL), thyroid-stimulating hormone (TSH), free thyroxine (FT4) and total testosterone (in males) from patient files. Women with regular menstrual cycles who did not use oral contraceptives were considered to have no gonadotropic axis deficiency. In post-menopausal women, the FSH levels were analyzed. We could not evaluate the presence of hypocortisolism in all the patients because some patients were transferred from other centers and were being treated with high doses of dexamethasone or hydrocortisone before admission to our center or were using oral corticosteroids. Hypopituitarism was defined as the presence of at least one endocrine axis.

We collected laboratory results reported at admission and at the last evaluation at IECPN until September 2017. The laboratory tests recorded at admission were performed, in cases, outside IECPN, at different laboratories.

### Radiological evaluation

All the patients had at least one MRI described in the medical records confirming PA. Twenty of the 35 patients had undergone MRI at our center, and these MRI images were available in our database system. The MRIs of these 20 patients were reviewed by the same experienced neuroradiologist that analyzed the sagittal and coronal T1-weighted images (T1WI), with and without gadolinium contrast, and coronal T2-weighted images (T2WI).

Microadenomas were defined as those with a maximum diameter < 10 mm, and macroadenomas were defined as those with a maximum diameter ≥ 10 mm (30). The tumor volume was estimated using the DiChiro and Nelson formula (width × height × length × 0.5233) (31). Patients were grouped according to the MRI findings using the classification of typical stages of hematoma evolution in the brain, as shown in Table 1 (32).

**Table 1:** Stages of hematoma as observed on MRI

Stage	Time since apoplexy	Hemoglobin	T1WI	T2WI
Acute	≤ 7 days	Deoxyhemoglobin	Isointense or Slightly Hyperintense	Very Hypointense
Subacute	> 7 days to ≤ 21 days	Methemoglobin	Hyperintense	Hyperintense
Chronic	> 21 days	Hemosiderin	Hypointense	Hypointense

MRI: magnetic resonance imaging; T1WI: T1-weighted imaging; T2WI: T2-weighted imaging. Radiological classification of hematoma evolution on MRI (32).

### Screening for AIP mutations

We used the PureGene Blood Kit (Gentra, Minneapolis, MN, USA) to obtain genomic DNA from 300 μL of whole blood following the manufacturer's instructions. DNA was resuspended in 100 μL of DNA Hydration Solution (Gentra). After extraction, PCR was performed using an Applied Biosystem ProFlex™ PCR System (Thermo Fisher Scientific, Foster City, CA, USA).

Genomic analyses included exons 1 to 6 from the *AIP* gene and flanking intronic sequences. Amplification and sequencing were performed using *AIP* PCR/Sanger Sequencing Primer pairs (Thermo Fisher Scientific™, Boston, MA). The reaction contained a mixture of 30 ng of genomic DNA, 2 U of Platinum^®^ Taq DNA Polymerase (Invitrogen, Foster City, CA, USA), 1.5 mM MgCl_2_ and 0.2 μM of each primer, with a total volume of 25 μL (Table 2).

**Table 2:** Primers used for *AIP* gene sequencing

PRIMERS				
Cat N°	Lot n°	Description/Sequence	Exon	Product size
A15633 – Hs00394559	292760 G02	TGTAAAACGACGGCCAGTCCGAGACATTCCTAGGCTCCG	1	495
A15634 – Hs00394559	292788 B02	CAGGAAACAGCTATGACCGCCCGAATTCACCCCCTACTTAAA		
A15633 – Hs00394560	292760 G03	TGTAAAACGACGGCCAGTGGAAGCCCCGTCCCTTATGC	2	381
A15634 – Hs00394560	292788 B03	CAGGAAACAGCTATGACCAGTCTAGCAGAGGGTGGAGGGAG		
A15633 – Hs00394561	292760 G04	TGTAAAACGACGGCCAGTCGGAGTAGGGTCCCAGTTGTC	3	492
A15634 – Hs00394561	292760 G07	CAGGAAACAGCTATGACCGGAGACCCAGGGTACTGCCAA		
A15633 – Hs00394562	292760 G05	TGTAAAACGACGGCCAGTCCAGATGTGGGTCAGGTCTGC	4	501
A15634 – Hs00394562	292788 B04	CAGGAAACAGCTATGACCGTCGTACTTGTTGAGGATGGAAGA		
A15633 – Hs00394563	292788 B01	TGTAAAACGACGGCCAGTAAGGTACTGCCTGGAGGCTGAG	5	507
A15634 – Hs00394563	292788 B05	CAGGAAACAGCTATGACCTCATGTCTCCTGGCACCATGGG		
A15633 – Hs00394564	292760 G06	TGTAAAACGACGGCCAGTGTGGCATCCTCAGGTCAGGGA	6	509
A15634 – Hs00394564	292788 B06	CAGGAAACAGCTATGACCGTACCAGGAATGCCAGGTGATGAC		

AIP: aryl hydrocarbon receptor-interacting protein.

PCRs followed an initial denaturation and enzyme activation at 94 °C/5 min and then 40 cycles of denaturation at 94 °C/45 sec, annealing at 94 °C/45 sec and extension at 72 °C/1 min. A final extension was performed at 72 °C for 7 min. PCR product clean-up was performed using the ExoSAP-IT^®^ system (USB Corporation, Cleveland, OH, USA), and DNA sequencing using the Big Dye Terminator v3.1 Cycle Sequencing kit (Thermo Fisher Scientific).

The products were sequenced in both directions on an ABI 3130xl Genetic Analyzer (Applied Biosystems), and electropherogram-derived sequences were aligned using Benchiling (https://benchiling.com/) and BioEditsoftware (http://www.mbio.ncsu.edu/BioEdit/bioedit.html). The reference sequences for the *AIP* gene used were as follows: ENSG00000110711 (https://www.ensembl.org/Homo_sapiens/Gene/Summary?g=ENSG00000110711;r=11:67483041-67491103), NG_008969.3 (https://www.ncbi.nlm.nih.gov/protein/NP_003968) and NM_003977.3 (https://www.ncbi.nlm.nih.gov/nuccore/NM_003977).

### Statistical analysis

SPSS version 23.0 for Windows was used for statistical analysis, and the data were presented as percentages, means ± standard deviation (SD) or medians (min-max). Normal distribution was tested, and the Mann-Whitney test was used to compare numerical variables between groups. A p-value < 0.05 was considered significant.

## RESULTS

### Demographical and tumor characteristics

Thirty-five patients were included (20 males), with a mean age of 40.5 ± 17.1 years. Non-functioning pituitary adenomas were present in 23 patients (66%), seven (20%) harbored somatotropinomas, and five (14%) harbored prolactinomas. Only three patients (9%) had a previous diagnosis of pituitary adenoma before apoplexy: two patients harboring NFPA and one acromegaly patient. Only the acromegaly patient had started treatment before the PA episode (octreotide LAR four months before). The others were treatment naïve.

### Clinical characteristics

Headache was the most common symptom, present in 31 patients (89%). Among these, 16 patients (52%) presented with a thunderclap headache, ophthalmoplegia was observed in 13 patients (37%), and six patients (17%) presented with ptosis. Ten (28%) patients presented with visual field defects, and changes in the level of consciousness were present in five (14%) patients. Five (14%) patients presented with ophthalmoplegia and visual loss concomitantly. At the last assessment, 9 (25%) patients persisted with some degree of visual field defects. No patient presented headache or a reduced level of consciousness at the last evaluation. The median period between admission and last evaluation was six months (ranging from 3 to 48 months). The clinical presentation data are summarized in Table 3.

**Table 3:** Clinical findings

Symptoms	At acute event N (%)	At last evaluation* N (%)
Headache	31 (89%)	0
Thunderclap headache	16 (46%)	0
Ophthalmological signs and symptoms&	18 (51%)	11 (31%)
Ophthalmoplegia	13 (37%)	03 (8%)
Ptosis	06 (17%)	02 (6%)
Visual field defect	10 (28%)	09 (25%)
Altered consciousness	05 (14%)	0

* Median follow-up: 6 months after the event (range: 3 to 48 months).

& Some patients presented more than one ophthalmological symptom.

### Laboratory characteristics

At admission, 19 patients (54%) had deficiency of at least one pituitary axis. Hypothyroidism was present in nine patients (26%), and hypogonadism was also present in nine patients. None of them were using hormonal replacement before the acute event. We identified GH deficiency in six of 21 patients with IGF-I available at the first evaluation. Prolactin levels were available from 20 patients, and eight (20%) patients had hyperprolactinemia. We observed worsening of hypothyroidism and hypogonadism at the last evaluation, with 17 (48%) and 16 (45%) patients presenting these deficiencies, respectively. The time elapsed from the acute PA event to the first laboratory evaluation varied from 3 days to 5 months. Considering the group of 10 patients treated surgically, three (30%) improved pituitary function completely, two (20%) presented worsening of the pituitary axis, and five (50%) persisted with the same pre-operative hormonal deficits.

### Predisposing factors

Potential predisposing factors were investigated, and no PA was found after cardiac surgery, radiotherapy, endocrinological function testing, and the use of anticoagulant medication or antiplatelet agents. Nine patients had arterial hypertension, and eight patients were using oral contraceptives; in one patient, the onset of PA occurred during intense exercise (running); in another patient, PA occurred four months after starting octreotide LAR treatment.

### Radiological characteristics

In our cohort, one microadenoma (9 × 8 × 7 mm) was found. The median tumor volume in the whole group was 5.4 cm^3^ (0.26-48.67 cm^3^), and the median larger tumor diameter was 2.9 cm (0.9-6.2 cm). Somatotropinomas exhibited larger tumor diameters and volumes than other tumor types. The median tumor volumes were 26.9 cm^3^ (5.64-48.67 cm^3^) and 4.5 cm^3^ (0.26-16.32) in acromegaly patients and other tumor types, respectively (p = 0.021). The median larger tumor diameters were 4.2 cm (1.0 to 6.2 cm) and 2.5 cm (0.9 to 4.5 cm) in somatotropinomas and other tumor types, respectively (p = 0.013). No significant difference was observed in the larger tumor diameter between groups with or without neuroophthalmological symptoms (p = 0.18).

Considering the 20 MRIs available in our database that were reviewed by our neuroradiologist, various signal intensities of the pituitary adenomas in both T1WI and T2WI were observed, regardless of the elapsed time since apoplexy (Table 4). The time elapsed from acute PA to MRI varied from 3 days to 5 months; most of the patients (17 patients) were in the chronic phase. No clear pattern of evolution of the hemorrhagic image was observed after the episode of PA. Seventeen patients in this group presented with acute PA events more than three weeks before the MRI scan had been performed; in two of them, PA occurred five months before. However, in eight patients, a hyperintense signal in T1WI was still present; in six patients, a heterogeneous pattern (some areas of hyperintense signals and other areas of hypointense signals in T1WI at the same time) was observed.

**Table 4:** Radiological findings

Stages of Hematoma*	ΔT symptoms and first MRI at IECPN	Tumor Type	T1	T2	Optic chiasma compression	High SI in T1	Surgery during the follow-up	ΔT from first to last MRI at IECPN	MRI findings at last MRI
≤ 7 days									
Acute	3 d	ACRO	Hyperintense	Hyperintense	Yes	Yes	Yes		
Acute phase	7d	NFPA	Heterogenous	Heterogenous	Yes	Yes	No
> 7 days and ≤ 21 days									
Subacute phase	20d	NFPA	Hyperintense	Hyperintense	Yes	Yes	Yes		
> 21 days Chronic	28 d	NFPA	Hyperintense	Hyperintense	No	Yes	No		
	28 d	NFPA	Hyperintense	Isointense	Yes	Yes	Yes		
	28 d	NFPA	Heterogeneous	Heterogeneous	Yes	Yes	No	3 m	Empty sella
	28 d	NFPA	Heterogeneous	Heterogeneous	Yes	Yes	Yes		
	30 d	NFPA	Isointense	Isointense	No	No	No	2 m	Heterogeneous
	30 d	NFPA	Hyperintense	Isointense	Yes	Yes	Yes		
	2 m	PRL	Hyperintense	Heterogenous	No	Yes	No		
	2 m	NFPA	Isointense	Hypointense	No	No	Yes		
	2 m	NFPA	Heterogeneous	Hyperintense	Yes	Yes	Yes		
	2 m	NFPA	Isointense	Heterogeneous	No	No	No	5 m	Heterogenous
	2 m	NFPA	Heterogeneous	Heterogeneous	Yes	No	No		
	3 m	ACRO	Isointense	Heterogeneous	No	No	Yes		
	3 m	PRL	Isointense	Isointense	No	No	No	7 m	Empty sella
	3 m	PRL	Hyperintense	Heterogeneous	No	Yes	Yes		
	3 m	PRL	heterogeneous	Heterogeneous	Yes	Yes	Yes		
	5 m	NFPA	Isointense	Heterogeneous	No	No	No	8 m	Empty sella
	5 m	NFPA	Hyperintense	Hypointense	No	Yes	No	8 m	Empty sella

MRI: magnetic resonance imaging; SI: signal intensity; ACRO: somatotropinoma; NFPA: non-functioning pituitary adenoma; PRL: prolactinoma; d: days; m: months; T1 and T2 classification ΔT: time elapsed from PA to examination.

**Figure 1:**
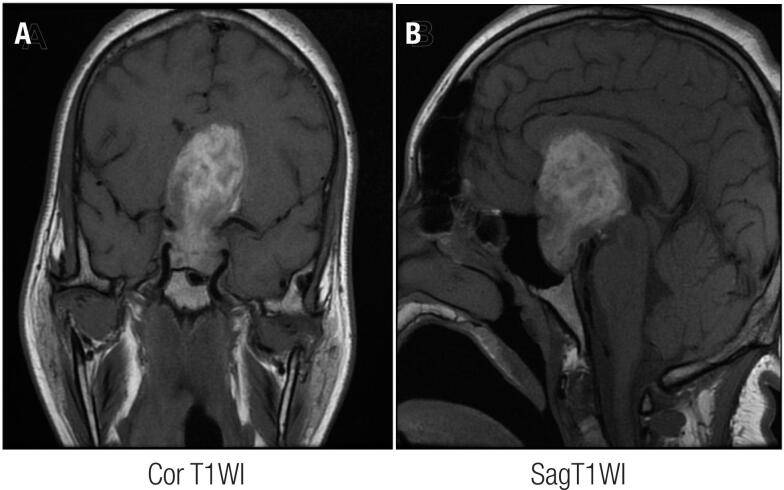
Coronal T1 (A) and sagittal T1-weighted imaging (B) sequences of the magnetic resonance imaging of a 16-year-old patient performed three days after the pituitary apoplexy event and showing a mass in the sellar region with important suprasellar and infrasellar extension, presenting hyperintense areas.

We compared the first and last MRI scans of six patients who were treated conservatively. Progression to an empty sella was verified in four patients, and two patients maintained the heterogeneous pattern in the T1WI described above. The interval from the first to last MRI of these six patients was 5 months on average, ranging from three to eight months. No re-bleeding was observed. These radiological findings are summarized in Table 4. Figure 1 illustrates the MRI of a patient in the acute phase of PA.

### Treatment characteristics

Seventeen (48%) patients did not present any visual field impairment, ophthalmoplegia or ptosis and were treated conservatively. Eight (23%) patients presented with only ophthalmoplegia and/or ptosis with no visual loss and received conservative treatment. All 10 (28%) patients who presented visual loss had undergone surgery. In this group visual recovery was complete in one patient (10%) and partial in three patients (30%), and no improvement was observed in the remaining six (60%) patients.

### *AIP* mutation screening

No *AIP* mutation was identified. We found four different single-nucleotide polymorphism (SNP) variants: two intronic variants [c.132C>T p. Asp44= in exon 2, c.516C>T p. Asp172= in exon 4] and two nonsynonymous variants [c.682 C>A and c.920 A>G] in exons 5 and exon 6 in 31 and 9 patients, respectively. The most frequent allelic variant (31 patients) was present in exon 5, c.682C>A, encoding Q228K. In exon 6, we found other nonsynonymous SNP variants, c.920A>G, encoding Q370R (nine patients) and one intron variant insertion (c.788-30_788-29ins-/TGCCCAC). All the variants observed were considered benign in the general population (33,34).

## DISCUSSION

In our cohort, acute onset of headache was the most common symptom of PA and was managed conservatively in patients without visual loss. Radiological evaluation showed that persistent high signal intensity in T1WI lasted longer than usually described in other areas of the brain. Additionally, we showed that *AIP*mut screening is likely not useful in these patients.

Male predominance was observed to be similar to most series. However, in our cohort, the mean age was 40.5 years, younger than that reported in the literature, in which patients presented most frequently in the 5^th^ or 6^th^ decade of life (3,9,35–40).

Headache was the most frequent symptom, and these results agreed with the literature, in which headache was present in 63%-100% of cases (29,37,40,41). Thunderclap headache was described in approximately 46% of patients with PA, and we observed similar results (51%), with no worsening evolution results in this group (29,37,40,41).

Neuroophthalmological symptoms and visual field defects were present in 51% (n = 18) of patients, including ptosis and ophthalmoplegia. These results were similar to the literature, in which a frequency of visual disturbance varying from 23% to 81% (3,42–44) and impairment of III, IV and VI cranial nerves of 52% was described (3,45).

Hypopituitarism is described in 50%-86% of all investigated patients (3,6,45). In our study, we observed a frequency of 54% at admission that progressed to 60% at the last evaluation. Similarly, other studies showed that improvement in pituitary function after PA, independent of treatment should not be expected (6,37,42,46). One limitation of our study was that we could not precisely estimate the frequency of hypocortisolism in our patients because many patients were admitted to other centers before being transferred to ours and were already using high doses of dexamethasone/hydrocortisone or oral corticosteroids at admission in our center. Additionally, the real frequency of GH deficiency could not be determined because we did not perform functional tests, such as the insulin tolerance test, in patients with normal IGF-I levels to ensure that they were not deficient.

The initiation or withdrawal of dopamine agonist; octreotide withdrawal; thrombolytic, anticoagulation and antiplatelet therapy; estrogen therapy; coagulopathies, dengue hemorrhagic fever; cardiac and other major surgeries; dynamic pituitary function tests; radiation therapy; pregnancy and postpartum state have been listed as potential apoplexy precipitating factors (1,9,12–14,43,47,48). In our study, nine patients (25%) had arterial hypertension, consistent with the results of other publications, indicating that this condition is a common feature in patients with PA (3,6,9,13,18). However, Möller-Goede and cols. (9) published a review with 574 patients and observed that arterial hypertension and diabetes mellitus did not increase the risk of PA, similar to that in a previous study by Biousse and cols. (13). One patient had syncope during intense exercise that might be related to an abrupt change in tumor vascular pressure, previously described in other conditions associated with an increase in blood pressure (23).

Magnetic resonance imaging is the most important radiological tool to study apoplexy, with sensitivity ranging from 80% to 90% (16). Typical MRI descriptions during the acute phase include areas of hyperintense signal on the pituitary region on T1WI (49,50). However, many descriptions of hematoma evolution after apoplexy are derived from what is observed in other parts of the brain, and a lack of specific studies exists to describe hematoma evolution in PA (30,49). Some single-center studies of PA have been reported, but imaging features were, in general, not detailed (2,36,37). Generally, PA imaging studies describe acute events without MRI at follow-up (15,44,50).

The most frequent radiological feature of PA in the literature is hyperintense signals on T1WI, but other conditions can present the same characteristics, such as aneurysms, lipomas and Rathke cleft cysts (RCCs) (32). T2WI can help in the differentiation of an intracystic hypointense nodule, a typical sign of RCC related to proteinaceous fluid (32,51).

The two most specific image patterns of PA, sphenoid sinus mucosal thickening and fluid debris or fluid-fluid level (hyperintense signal on T1WI in upper fluid), were not found in our series (15,52). The first pattern can appear even before the vascular event, suggesting an engorgement caused by large adenomas or large collections of blood and likely related to severity and generally observed in the acute phase (52). The second pattern is the fluid-fluid level due to free extracellular methemoglobin in the upper fluid layer and with blood residue in the lower layer and is mostly described in the subacute phase (49–52).

**Figure 2:**
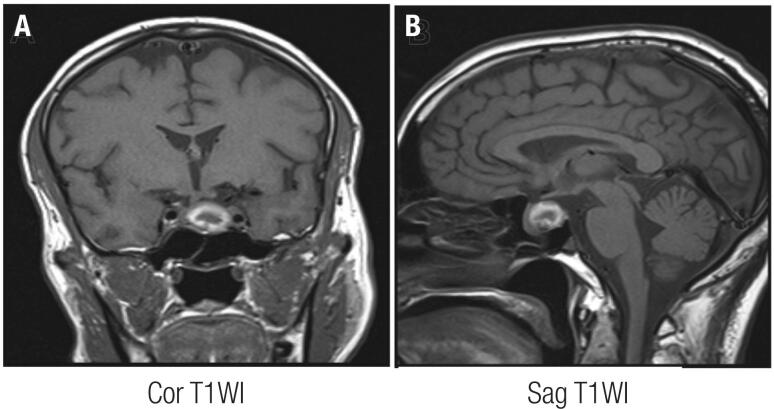
Coronal (A) and sagittal T1-weighted imaging (B) sequences of magnetic resonance imaging performed in a patient with classical pituitary apoplexy, 30 days after the acute event and showing a ring area of hyperintense signals in the sellar lesion.

A persistent hyperintense signal on T1WI was observed in our series even when MRI was performed in a later period, suggesting slower pituitary hematoma absorption. In vascularized areas of the central nervous system (subdural and epidural areas), oxygen tension remains high and slower from one stage to the next than in the brain itself (30,49). This condition can interfere with the evolution of hematoma and may explain our MRI results in patients outside the acute or subacute phase. Piotin and cols. (50) published an analysis of MRI patterns in PA and showed that, in general, high signal intensity, particularly hyperintensity in T1WI, suggests the presence of blood, but pituitary hemorrhage may present with a persistent hyperintense signal. We found a pituitary ring sign, typically described in the acute phase in a patient who had 30 days of evolution since the occurrence of PA symptoms (Figure 2 A; B) (52).

The risk of re-bleed can occur in a range from 6% to 11% of cases described in different studies, and the results are similar regardless of surgical or conservative approach (15,18,41,45,53). We found no apparent re-bleeding, and an empty sella was observed in four of six patients who had undergone a second MRI at IECPN. It took three to eight months after PA for the emergence of an empty sella, suggesting a longer time for hematoma absorption on pituitary topography.

The management of patients with PA can be surgical or conservative, and several retrospective studies have shown that the results are similar. In particular, conservative management is performed in patients with mild symptoms and stable ophthalmological deficits (6,37,40,42). Many studies have demonstrated spontaneous resolution of visual and neurological symptoms with expectant management (54–57). However, until now, no randomized trial has compared both strategies (35,36,39). The severity of symptoms at presentation and presence and progression of visual impairment should be considered parameters to guide better treatment for these patients (6,18,37,40). We initially treated 25 patients without visual impairment conservatively despite other neuroophthalmological symptoms (ptosis and/or ophthalmoplegia) with good outcomes.

Some series suggest that apoplexy may be a clinical feature of patients with *AIP* mutations, as noted by Igreja and cols. (20), who described apoplexy in 8% of familial series. This mutation is associated with larger tumors with aggressive behavior and a young onset (21,22,58). In our series, *AIP* screening revealed no mutation (33,59).

Two nonsynonymous SNP variants that promote amino acid substitutions (Q228K and Q307R) were described as missense variants with increased prevalence in FIPA patients (34). In the same study, sporadic forms of Cushing's disease were also associated with a variant of Q307R, and sporadic acromegaly was associated with a variant of Q228K (34). Both allelic variants have already been described in some subpopulations with moderate frequency (59). However, until now, no functional study was performed to confirm the relevance or pathogenicity of these allelic forms, and no other study found an association with FIPA or any other familial disease (59).

Recently, a large study with 2,227 patients analyzed variables that could help in *AIP* screening and, indeed, apoplexy was more frequent in *AIP*mut patients. The mechanisms by which *AIP* mutations may lead to apoplexy may be related to rapid cell growth and proliferation (3,24). However, after multivariate analysis, it was not one of the variables that helped predict patients with an *AIP*mut (60). Most of the patients included in this study had a family history of pituitary adenomas. In our study, which included only patients with sporadic pituitary adenomas, we also found no *AIP* mutations, indicating that it is likely not valuable to perform *AIP* mutation screening in patients with PA.

In conclusion, apoplexy is associated with neuroophthalmological symptoms in a great proportion of patients but can be managed conservatively in selected cases. Images of PA on MRI may not present the classic evolution described for hemorrhagic events in other areas of the central nervous system, with the persistence of areas of hyperintense signals on T1WI after the acute and subacute phases. *AIP* mutations are not common, and *AIP* screening should not be performed in the absence of other features suggesting the presence of this mutation.
